# The global landscape of SCD and approach to case work-up in Latin America: a LAHRS and SOBRAC survey

**DOI:** 10.1007/s10840-026-02393-9

**Published:** 2026-07-10

**Authors:** Luciana Sacilotto, Alejandro Cuesta, Bruno Moreira, María José Arocena, Mariana Carvalho, Alexsandro Fagundes, Cristiano Pisani, José Eduardo Krieger, Francisco Darrieux, Nestor López-Cabanillas, Maurício Scanavacca

**Affiliations:** 1 Instituto do Coração, HCFMUSP, Av. Dr. Enéas Carvalho de Aguiar, 44 - Cerqueira César, São Paulo, SP 05403-900 Brasil; 2 Unidad Académica Cardiología, Universidad de la República, MUCAM, Montevideo, Uruguay; 3 Instituto de Cardiologia Integral, MUCAM, Montevideo, Uruguay; 4 Instituto Cardiovascular Adventista, Buenos Aires, Argentina

**Keywords:** Sudden cardiac death, Out-of-hospital cardiac arrest, Cardiogenetics, Molecular autopsy, Genetic testing, Latin America

## Abstract

**Background:**

Sudden cardiac death (SCD) remains a major global public health problem, yet data from Latin America are scarce. The region accounts for nearly 660 million people (approximately 8% of the world’s population), is highly admixed, and remains underrepresented in randomized controlled trials and cardiogenetic biobanks. Limited evidence exists on the epidemiology, diagnostic approaches, and use of cardiogenetic evaluation or molecular autopsy after unexplained SCD.

**Methods:**

We conducted a descriptive cross-sectional survey, endorsed by the Latin American Heart Rhythm Society (LAHRS) and the Brazilian Society of Cardiac Arrhythmias (SOBRAC). The questionnaire (20 closed questions, available in Portuguese, Spanish, and English) was distributed through society mailing lists and local physician networks between March 24 and April 12, 2025. General practitioners, intensivists, and cardiologists practicing in Latin America were eligible. Responses were anonymous, and surveys with < 80% completion were excluded.

**Results:**

A total of 439 physicians from 11 countries responded, most from Brazil (83.6%). Time in practice was heterogeneous: 38.7% reported < 10 years, 26.7% 10–20 years, and 34.6% >20 years. Only a minority considered regional out-of-hospital cardiac arrest (OHCA) systems efficient; 82% reported deficiencies in response times, training, and resources. The availability of automated external defibrillators (AEDs) in public spaces was reported as very limited (5.9%), and 88% indicated that BLS/ACLS educational initiatives were insufficient. Cardiogenetic infrastructure was also scarce: only 32 respondents (7.3%) reported access to specialized centers with counseling and testing, while 35% indicated genetic testing was available only in private settings. Familiarity with molecular autopsy was limited to 53.8%. Only 22% would order genetic testing after unexplained SCD, while 30.8% reported a lack of access or experience. In cases of unexplained SCD < 40 years, 76.5% reported screening relatives clinically, 49.2% would consider genetic testing of relatives, and 39.6% would seek post-mortem samples. Importantly, 85.2% of respondents expressed willingness to participate in a multicenter genetic protocol for SCD in young individuals.

**Conclusion:**

This first multinational survey provides a unique overview of SCD work-up in Latin America, highlighting critical gaps in OHCA response, cardiogenetic infrastructure, and molecular autopsy availability. Despite limited resources, there is strong interest in collaborative efforts, underscoring the urgent need for region-specific policies, multicenter protocols, and educational strategies to improve SCD care and prevention in Latin America.

**Graphical Abstract:**

**Figure:**
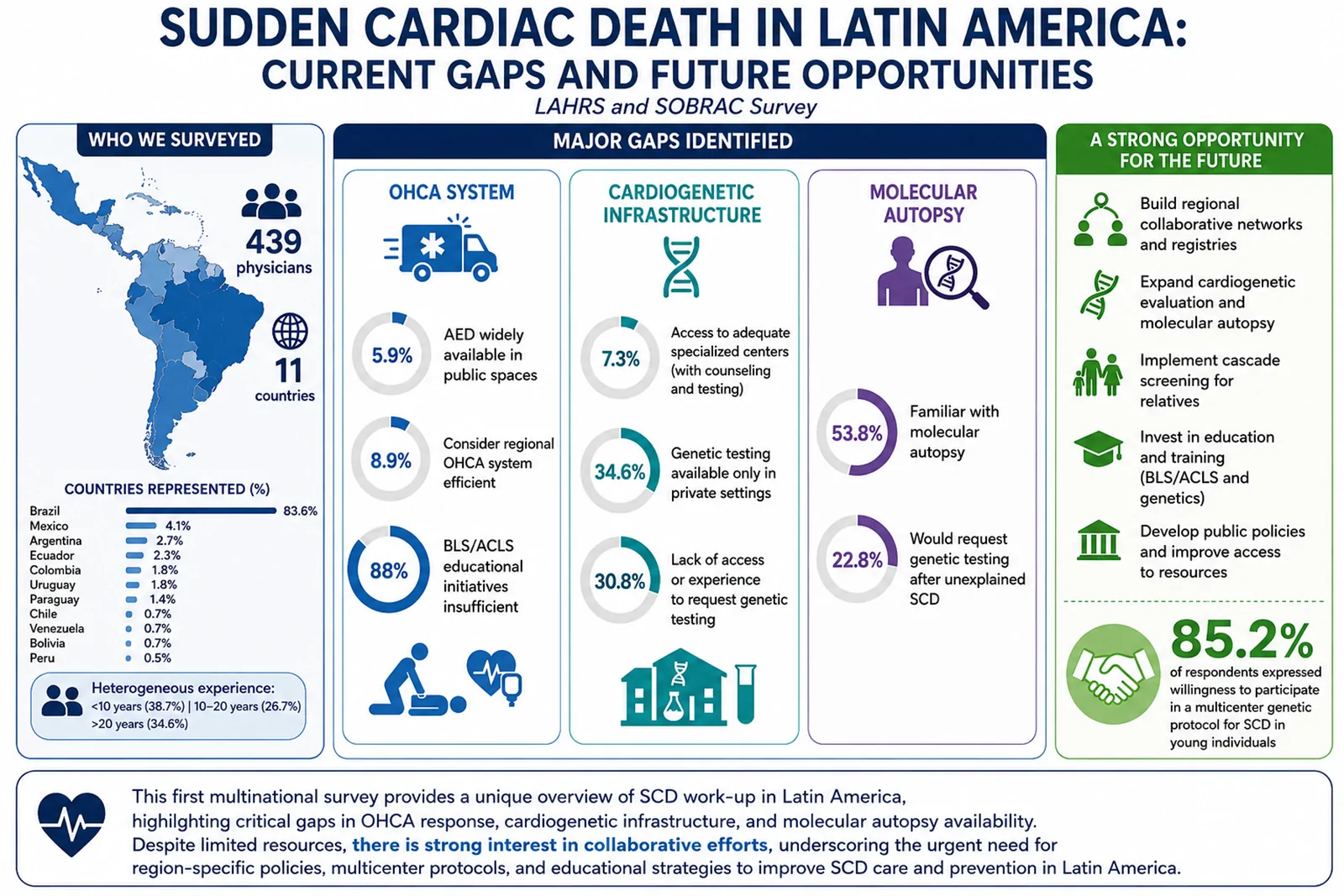
Central Illustration. Sudden Cardiac Death in Latin America: Current Gaps and Future Opportunities — Findings From the LAHRS and SOBRAC Survey.A multinational survey encompassing 439 physicians across 11 Latin American countries, predominantly from Brazil (83.6%), with representation from Mexico, Argentina, Ecuador, Colombia, and others, was conducted to characterize the current landscape of sudden cardiac death (SCD) workup and identify critical structural gaps across three domains (left panel). Major deficiencies were identified in out-of-hospital cardiac arrest (OHCA) systems, cardiogenetic infrastructure, and molecular autopsy availability (central panels). Regarding OHCA response, only 5.9% of respondents reported widespread AED availability in public spaces, 8.9% considered regional OHCA systems efficient, and 88% deemed BLS/ACLS educational initiatives insufficient. In the cardiogenetic domain, access to specialized centers with genetic counseling and testing was reported by only 7.3% of participants, genetic testing was available exclusively in private settings according to 34.6%, and 30.8% lacked experience or access to request genetic testing. Concerning molecular autopsy, only 53.8% were familiar with the concept, and a mere 22.8% would request genetic testing following an unexplained SCD. Despite these limitations, 85.2% of respondents expressed willingness to participate in a multicenter genetic protocol for SCD in young individuals, underscoring strong regional interest in collaborative research (right panel). Future priorities include building regional registries, expanding cardiogenetic evaluation and molecular autopsy programs, implementing cascade screening for relatives, and developing region-specific public policies to improve SCD prevention and care across Latin America.AED = automated external defibrillator; ACLS = advanced cardiovascular life support; BLS = basic life support; LAHRS = Latin American Heart Rhythm Society; OHCA = out-of-hospital cardiac arrest; SCD = sudden cardiac death; SOBRAC = Sociedade Brasileira de Arritmias Cardíacas.

## Introduction

Sudden cardiac death (SCD) remains a major global public health problem, accounting for an estimated 4–5 million deaths annually [1]. Although the epidemiology and prevention strategies for SCD have been extensively studied in North America and Europe, there is a significant lack of data from low- and middle-income regions, including Latin America and the Caribbean, which together represent nearly 660 million people, approximately 8% of the world’s population.

The population in this region is highly mixed-race and remains underrepresented in randomized controlled trials and cardiogenetic biobanks, limiting the generalizability of current evidence. In Latin America, available data on out-of-hospital cardiac arrest (OHCA) reveal important system-level challenges [2–4]. In a metropolitan area in Southern Brazil, only 2.4% of emergency calls were attended within the optimal 5-minute response window. Conversely, local initiatives demonstrate that improvements are possible: in the São Paulo metro system, where security officers are trained in cardiopulmonary resuscitation, bystander CPR was performed in 100% of cases, with a mean collapse-to-defibrillation time of 4.8 ± 1.2 min [5]. These findings highlight both the barriers and opportunities for improving SCD outcomes in the region.

Diagnostic approaches for SCD in Latin America, particularly in the context of cardiogenetic evaluation and molecular autopsy, are scarce. Only a limited number of studies from Latin America have been published, and the natural history of cardiogenetic diseases in this population remains poorly characterized [2–4]. Genetic testing continues to be underused across the region, reflecting barriers in access, awareness, and implementation. Even less is known about the role of molecular autopsy in cardiovascular diseases, with virtually no systematic data available, except small retrospective studies [6]. This scarcity of evidence underscores the need for region-specific investigations to guide clinical practice and policy.

To address this gap, the Latin American Heart Rhythm Society (LAHRS) and the Brazilian Society of Cardiac Arrhythmias (SOBRAC) conducted a multinational survey among physicians practicing in the region. This study provides an overview of clinical practice patterns and approaches to SCD case work-up in Latin America, with a particular focus on molecular autopsy, aiming to identify existing gaps and opportunities for future strategies.

## Methods

A descriptive cross-sectional observational study was done, using a closed questions survey, through an online commercial platform (SurveyMonkey^®^). The form was reviewed and endorsed by the Latin American Heart Rhythm Society and the Brazilian Society of Cardiac Arrhythmias. Questions were made on an individual basis and collected anonymously, only country of origin was recorded. Participants could leave responses blank, except country of origin. If less than 80% of the questionnaire was answered, that survey was not included in the analysis.

### Population

General practitioners, intensivists, and cardiologists working in Latin America were included, regardless of whether they were affiliated with electrophysiology services, performed invasive electrophysiological procedures (IEP), or their level of training. The call was made through the mailing list of active members of the Latin American Heart Rhythm Society (LAHRS) and Brazilian Heart Rhythm Society (SOBRAC). The call was also made through local lists of members. LAHRS or SOBRAC membership was not required to participate. Its participation and completion were not mandatory nor were encouraged by any economic and/or material good. The same survey had a Portuguese, English and Spanish version that were merged later. It was available to be answered 20 days, between March 24 and April 12, 2025.

The survey consisted of 20 questions in one block (supplement material). It included closed questions with a single, exclusive answer and answers with non-exclusive options. The survey did not provide patient information, and participants were asked for consent for the inclusion, analysis and presentation of the information.

The analysis was performed with Microsoft^®^ Excel^®^ 2016 MSO. The qualitative variables are expressed in absolute number and percentages. No comparisons were made.

## Results

### Population characteristics

**Fig. 1:**
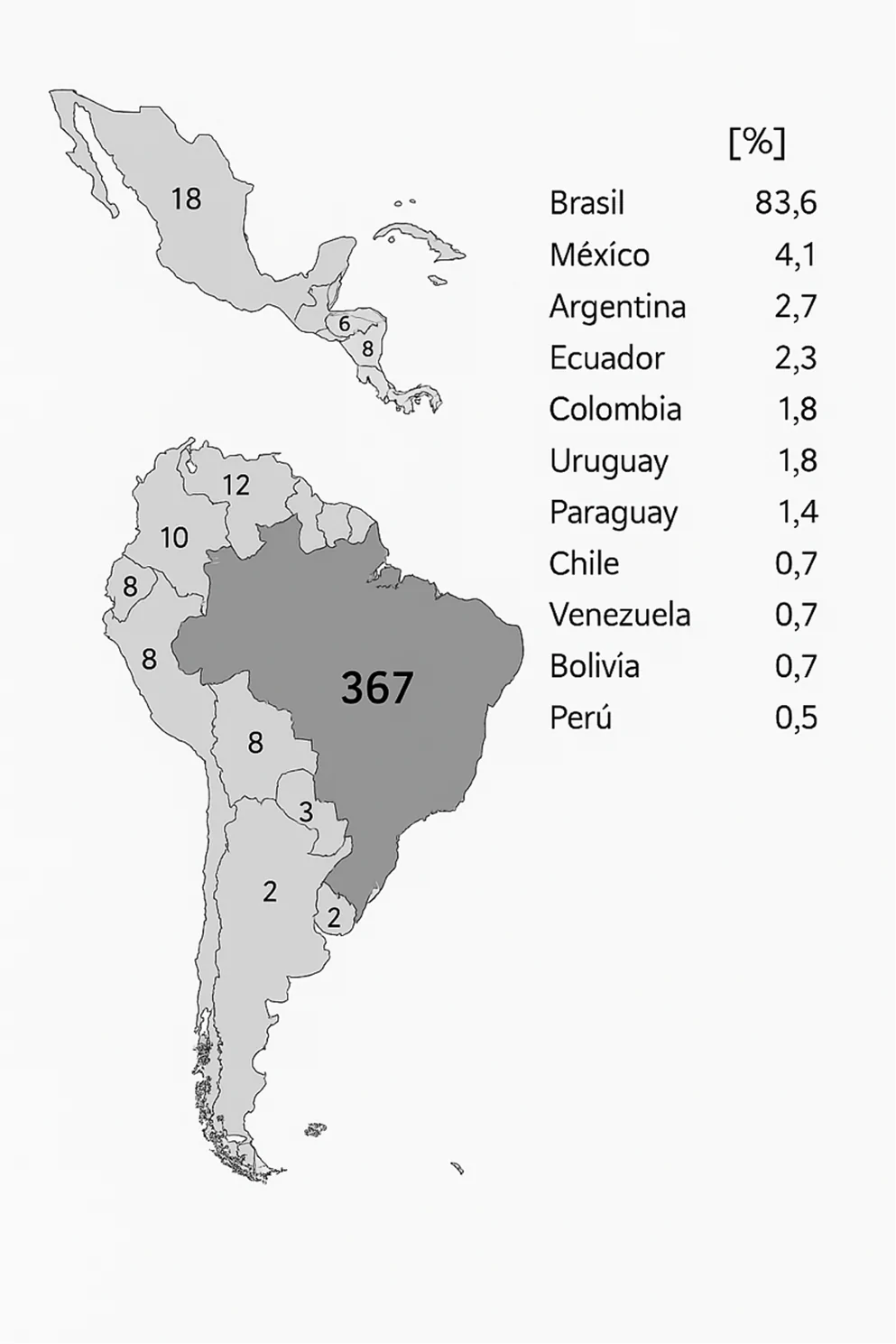
Latin American countries completed the survey

A total of 439 physicians from 11 Latin American countries completed the survey. Most respondents practiced in Brazil (*n* = 367; 83.6%), followed by Mexico (18; 4.1%), Argentina (12; 2.7%), Ecuador (10; 2.3%), Colombia (8; 1.8%), Uruguay (8; 1.8%), Paraguay (6; 1.4%), Chile (3; 0.7%), Venezuela (3; 0.7%), Bolivia (2; 0.5%), and Peru (2; 0.5%), as shown in Fig. 1. Time in practice was distributed as follows: 38.7% reported < 10 years, 26.7% had 10–20 years, and 34.6% had > 20 years of experience. Self-reported areas of practice included clinical cardiology (61.5%), electrophysiology, and pacemakers (25.1%), clinical arrhythmology (23.0%), echocardiography (8.2%), and interventional cardiology (1.8%); 18.0% identified as other than cardiologist (Fig. 1). Key gaps identified in the survey are summarized in Table 1.

**Table 1:** Key gaps in OHCA response and cardiogenetic infrastructure in Latin America

Answer	Key finding	Percentage
AED availability	AEDs widely available and well-maintained	5.9%
BLS/ACLS training	Training opportunities limited or absent	88%
OHCA response system	Physicians considering the system adequate	8.9%
Cardiogenetic infrastructure	Regions with sufficient specialized centers	7.3%
Molecular autopsy knowledge	Physicians unfamiliar with the concept	46.2%
Practical access to genetics	No experience or no access to services	30.8%

*OHCA* out-of-hospital cardiac arrest, *AED* automated external defibrillator, *BLS* basic life support, *ACLS* advanced cardiac life support

## Out-of-hospital cardiac arrest (OHCA) response and resources

When asked about the adequacy of the response to OHCA in their region, only a minority of physicians considered the emergency system efficient and well-equipped (8.9%). Most respondents highlighted important limitations (45.1%), reported either partial deficiencies related to response times and training, or significant gaps in emergency response and resources (36.7%). A small proportion of participants stated they were not familiar with the OHCA response in their region (9.3%).

Most respondents indicated that educational initiatives to train healthcare professionals and the general public in basic life support (BLS) and advanced cardiac life support (ACLS) were insufficient. Only a minority reported that training programs are widely available, while 88% stated that opportunities are either infrequent or very limited. A small proportion of participants reported being unaware of any such programs in their region.

The availability of automated external defibrillators (AEDs) in public spaces was reported to be very limited. Only 26 respondents (5.9%) indicated that AEDs are commonly found and well-maintained. The majority, 230 respondents (52.4%), reported that there are some AEDs but they are not widely distributed. A further 172 respondents (39.2%) stated that AEDs are rarely found in public areas, while 11 respondents (2.5%) were unsure about their availability in their region. When asked about barriers to improving survival after OHCA, 287 physicians (65.4%) considered that all the listed barriers contribute simultaneously to poor outcomes. Among specific factors, 99 respondents (22.5%) highlighted lack of public awareness and CPR training, 24 respondents (5.5%) pointed to delayed emergency medical response times, and 22 respondents (5.0%) cited the limited availability of AEDs in public spaces. Only 7 respondents (1.6%) reported that poor coordination between emergency services and hospitals was the main barrier.

## Cardiogenetic infrastructure and molecular autopsy

Access to cardiogenetic evaluation across the region was reported to be very limited. Only 32 respondents (7.3%) indicated the existence of sufficient specialized centers with access to both genetic counseling and testing. The most frequent response was that few specialized cardiogenetics centers exist, with limited access to these services (46.5%). In addition, 34.6% reported that genetic testing is available only in private settings, with little or no access through the public healthcare system. A further 11.6% indicated that, although genetic testing may be available, there is no structured cardiogenetics service to integrate results into clinical practice.

Knowledge about molecular autopsy was limited among respondents. Slightly more than half (53.8%) reported being familiar with the concept, while 46.2% stated that they did not know what molecular autopsy means. In the setting of unexplained sudden cardiac death (SCD), genetic testing practices varied widely among respondents. 100 physicians (22.8%) stated that they would request genetic testing in all cases, regardless of clinical context or victim’s age. 87 respondents (19.8%) would consider testing only if there was a family history of SCD or known genetic conditions, and 93 respondents (21.2%) would order testing only if the autopsy was suggestive of an inherited cardiac disease, or if it was inconclusive but suspicion remained for channelopathies or concealed cardiomyopathies. A considerable number, 135 respondents (30.8%), reported that they were not experienced with this approach and had no cardiogenetics services available in their region. Finally, a small minority, 24 respondents (5.5%), indicated that they would never request genetic testing in such cases.

In the scenario of unexplained SCD in a patient younger than 40 years, the most common approach was clinical screening of family members (336; 76.5%). In addition, 216 (49.2%) would consider genetic testing of relatives based on clinical findings, 174 (39.6%) would seek to obtain post-mortem samples, and 102 (23.2%) would proceed with genetic testing of family members regardless of clinical context.

When asked about potential participation in a multicenter protocol for the genetic study of SCD in young individuals, most respondents expressed interest: 374 (85.2%) stated they would be willing to participate.

## Discussion

This multinational survey by LAHRS and SOBRAC uncovers substantial gaps in the clinical approach to sudden cardiac death (SCD) in Latin America.

## Out-of-hospital cardiac arrest (OHCA) response and resources

The deficiencies in out-of-hospital cardiac arrest (OHCA) response - highlighted by limited training, scarce AED presence, and multiple systemic barriers - mirror global findings that timely bystander CPR and early defibrillation can dramatically improve survival, yet these interventions remain underutilized even in high-income regions [7]. In contrast, public access defibrillation programs in countries such as Denmark, Japan, and several states in the United States have achieved wide AED distribution and demonstrated survival benefits [8], while in our survey, only 5.9% of respondents indicated AEDs are commonly found and well-maintained, and nearly 40% reported they are rarely available.

These findings are consistent with data from Mexico highlighting the absence of structured OHCA systems, with limited coordination and lack of standardized registries. Furthermore, the underrepresentation of Latin America in global cohorts, such as the inclusion of only one country in international registries of idiopathic ventricular fibrillation, reinforces the urgent need for region-specific data [9, 10]. Supporting this perspective, international initiatives such as the Inter-American Alliance Against Sudden Cardiac Death have also highlighted major gaps in cardiac arrest systems across Latin America, including limited access to AEDs, insufficient public training, and the absence of coordinated registries. Collectively, these findings indicate that poor outcomes in the region are largely driven by system-level deficiencies rather than disease-specific factors [11]. 

## Cardiogenetic infrastructure and molecular autopsy

Cardiogenetic evaluation and molecular autopsy represent critical but underdeveloped elements of the SCD work-up in Latin America. Internationally, molecular autopsy has been recognized as a valuable forensic adjunct in sudden unexplained deaths, enabling identification of inherited arrhythmogenic syndromes and guiding cascade screening of relatives [12, 13]. 

Importantly, Latin America is one of the most genetically admixed populations in the world, with ancestry contributions from European, Indigenous, and African roots [14] this complexity has been well documented in genomic studies [15]. Thus, the underrepresentation of such populations in cardiogenetic biobanks and randomized controlled trials limits the applicability of current risk models and underscores the urgent need for region-specific data.

In high-income countries such as the United States, 85–90% of cardiology professionals report access to a genetics specialist, while in parts of Europe, nearly a thousand genetic counselors practice across 19 countries, supported by public or private systems [16]. In our survey, only 7.3% of respondents reported adequate access to genetic counseling and testing infrastructure, and over one-third indicated such testing was confined to private settings. These disparities reflect both healthcare system limitations and socioeconomic inequities, echoing broader patterns of racial and ethnic disparities observed in cardiovascular care globally.

Regarding molecular autopsy, our findings resonate with those of a recent global survey on cardiogenetic evaluation after SCD in the young, which demonstrated substantial heterogeneity in access to genetic testing, autopsy practices, and multidisciplinary services worldwide. Similar to our Latin American data, the global study identified limited availability of genetic counseling and systematic molecular autopsy, particularly outside high-income countries. Notably, while some European and North American centers reported structured cardiogenetic pathways, such infrastructure was virtually absent across Latin America [17]. 

Although 46.2% of respondents reported being unfamiliar with the concept of molecular autopsy, clinicians are still faced with the challenge of managing relatives of victims who did not survive. In many cases, the cause of death remains inconclusive—sometimes due to the absence of a conventional autopsy and almost always because molecular autopsy was not performed. In this scenario, 23.2% of respondents stated that they would proceed with genetic testing of family members regardless of clinical context. However, such hypothesis-free genetic testing in relatives after unexplained SCD is often requested with limited awareness that it is actually considered a class III indication in current guidelines [12]. 

## Conclusion

This multinational LAHRS–SOBRAC survey highlights critical gaps in the approach to sudden cardiac death in Latin America, including limited training, scarce AED availability, and underdeveloped cardiogenetic infrastructure. Molecular autopsy is rarely implemented, and genetic testing practices remain heterogeneous, often not aligned with guideline recommendations. Despite these barriers, the strong willingness of physicians to participate in multicenter protocols represents a unique opportunity to develop regional collaborations that can generate robust evidence and improve outcomes for families affected by SCD.

### Limitations

The main limitation of this study is that the sample of physicians surveyed was not taken randomly, nor by any other method that could guarantee the representativeness of the universe studied. This also limits the external validity of any subgroup comparison that might be considered.

## Future directions

1. Policy advocacy: Encourage governments to adopt AED legislation and invest in widespread BLS/ACLS training for both professionals and laypersons.
2. Infrastructure strengthening: Develop publicly accessible cardiogenetic services and integrate molecular autopsy into forensic protocols.
3. Regional collaboration: Build multicenter research networks to standardize SCD investigations and enable cascade genetic screening programs.
4. Education and awareness: Train clinicians and stakeholders on the clinical utility of molecular autopsy, variant interpretation protocols, and multidisciplinary follow-up guidelines.

## Data Availability

No datasets were generated or analysed during the current study.
